# Bronchopulmonary Fistula Development in an Elderly Male With COVID-19 Infection

**DOI:** 10.7759/cureus.31686

**Published:** 2022-11-20

**Authors:** Aldin Malkoc, Harpreet Gill, Natalie Liu, Danny T Nguyen, Alexander T Phan, Alexandra Nguyen, Bruce Toporoff

**Affiliations:** 1 Department of General Surgery, Arrowhead Regional Medical Center, Colton, USA; 2 Department of General Surgery, Kaiser Permanente Fontana, Fontana, USA; 3 Department of Surgery, California University of Science and Medicine, Fontana, USA; 4 Department of Internal Medicine, Arrowhead Regional Medical Center, Colton, USA; 5 Department of Cardiothoracic Surgery, Loma Linda University Medical Center, Loma Linda, USA; 6 Department of Cardiothoracic Surgery, Arrowhead Regional Medical Center, Colton, USA

**Keywords:** elderly population, geriatric surgery, pneumothorax (ptx), video-assisted thoracic surgery, bronchopulmonary fistula, covid-19 respiratory failure

## Abstract

COVID-19 pneumonia can cause a wide range of complications including pneumothorax and empyema. However, in severe cases, it can lead to bronchopulmonary fistula (BPF) formation and a persistent air leak due to a connection between the pleural space and the bronchial tree. We report the case of a 77-year-old man with a history of hypertension, who presented to the emergency department for evaluation of dyspnea. Admission labs were significant for a positive rapid antigen SARS-Cov-2 test and elevated troponin I. A chest x-ray demonstrated patchy interstitial opacification and ground glass appearance bilaterally. Within the first 24 hours of presentation, the patient developed a right-sided spontaneous pneumothorax and had a 14 French pigtail catheter placed. The patient subsequently developed a persistent air leak after chest tube placement and required video-assisted thoracoscopic surgery (VATS) with talc pleurodesis and a 32 French chest tube placement. In this unique case, we describe an elderly patient’s experience of bronchopulmonary fistula formation as a complication of COVID-19 pneumonia and the successful management of this complication with VATS.

## Introduction

The SARS-CoV-2 respiratory virus has various documented complications including cardiac injury, acute kidney injury, and liver dysfunction [1]. The virus has much higher fatality rates in individuals with underlying medical conditions such as hypertension, diabetes, and cardiovascular disease. Patients that are infected with SARS-CoV-2 have been documented to have significant immune responses from a cytokine storm, often resulting in acute respiratory distress syndrome [2]. The most commonly documented complication of COVID-19 pneumonia is barotrauma, including pneumothorax, pneumomediastinum, and subcutaneous emphysema. Reports suggest the incidence of barotrauma to be up to 40% in patients requiring invasive ventilation, and up to 8.1% in patients requiring non-invasive ventilation. Furthermore, barotrauma has been associated with up to 60% increased mortality in this patient population [3].

In severe cases of COVID-19 pneumonia, there have been several documented cases of bronchopulmonary fistula (BPF) formation. The development of an abnormal communication between the pleural space and the bronchial tree has been shown to have a morbidity of greater than 70% when left untreated [4]. Little research has been performed on bronchopulmonary fistula development and treatment in the COVID-19 patient population. In this case study, we present a case of a spontaneous pneumothorax in a SARS-CoV-2-positive, vaccinated 77-year-old male that did not resolve with appropriate interventions. His clinical course was complicated by the development of a BPF that required treatment with video-assisted thoracoscopic surgery with chemical pleurodesis.

## Case presentation

A 77-year-old male with a past medical history of hypertension presented to the emergency department with a three-day history of worsening rhinorrhea, cough, ageusia and anosmia, anorexia, headache, and generalized weakness. He was noted to be afebrile with a temperature of 97.0 °F, normotensive with a heart rate of 90, and hypoxic with peripheral oxygen saturation (SpO2) of 83% on room air. The patient was placed on high-flow nasal cannula with fractional inspired oxygen of 100% at 70L/min flow rate, which improved his oxygen saturation to 91-93%. On physical exam, the patient demonstrated increased work of breathing, and he was noted to have crackles and decreased breath sounds bilaterally on auscultation. The patient admitted to a sick contact at home with a dry cough. He had also completed a two-dose vaccination series with the Moderna COVID-19 vaccine.

**Figure 1:**
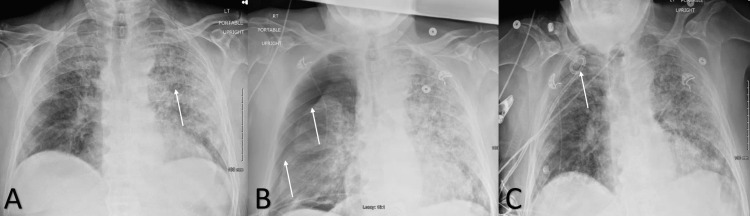
Anterior-Posterior chest x-ray on admission A). Anterior-Posterior chest x-ray on admission showing diffuse ground glass and interstitial opacifications, worse on the left side as indicated by the white arrow. B). Anterior-Posterior chest x-ray showing worsening left-sided ground glass and interstitial opacifications and a right-sided pneumothorax as shown by the white arrows. C). Anterior-Posterior chest x-ray with resolution of right-sided pneumothorax after placement of a 14 French pigtail catheter, as shown by the white arrow in the apex of the chest.

The complete blood cell count (CBC) showed leukocytosis of 11,900 cells/μL (Normal = 4,300-11,000 cells/μL) and hemoglobin of 12.8 g/dl (Normal = 13-17 g/dl). A basic metabolic panel was within normal limits except for a bicarbonate level of 20 mEq/L (normal = 24-34 mEq/L), blood urea nitrogen of 50 mEq/L (Normal = 8-20 mEq/L), and creatinine of 1.4 mg/dL (Normal = 0.7 to 1.3 mg/dL). Other laboratory findings were notable for serum lactate dehydrogenase of 2.6 mmol/L, ferritin of 865.6 ng/mL, and elevated Troponin I of 0.46 ng/ml. An electrocardiogram was performed and showed non-specific ST segment and T-wave changes. Coagulation tests were within normal limits. A urine drug screen (Roche Diagnostics Indianapolis, IN) was negative. The patient’s Influenza Type A and B polymerase chain reaction (PCR) test was negative and the patient’s nasopharyngeal real-time reverse transcriptase PCR test for SARS-CoV-2 RNA was positive. The initial chest X-ray, as shown in figure 1A, showed diffuse ground glass and interstitial opacifications. The patient was admitted for acute hypoxic failure secondary to suspected COVID-19 pneumonia and acute NSTEMI.

The initial treatment for the patient included an intravenous (IV) heparin infusion with a partial thromboplastin time (PTT) goal of 90-120 seconds, oral aspirin 81mg daily, oral atorvastatin 40mg daily, oral dexamethasone 6mg daily for ten days, IV Remdesivir 100mg daily for five days, and oral baricitinib 4mg daily 7 days. After the first 24 hours, it was evident the patient had a Type 2 myocardial infarction that was noted with three negative electrocardiograms along with down-trending troponin I levels, and as such the heparin infusion was discontinued. An echocardiogram was done to rule out cardiac abnormalities and it was unremarkable for structural and functional abnormalities. On hospital day 3, due to the patient developing new dyspnea and shortness of breath, a chest x-ray was ordered, demonstrating spontaneous development of a right pneumothorax, shown in figure 1B. A right-sided 14 French pigtail catheter was placed in the right hemithorax for lung re-expansion, shown in figure 1C.

On hospital day 6, the patient's clinical condition appeared to stabilize and he was weaned from high flow nasal cannula to 5L of low-flow nasal cannula oxygen supplementation. A chest tube clamp trial was performed, and the patient quickly decompensated with new-onset tachypnea, tachycardia, shortness of breath, and oxygen desaturation to 80%. A chest x-ray showed a worsening right-sided pneumothorax, so the chest tube was placed back to suction. At this time, the patient required 100% fraction of inspired oxygen on 70L/min high-flow nasal cannula.

On hospital day 9, it was evident that the patient was withdrawing from participation in daily activities. He experienced generalized weakness, poor appetite, and endorsed feelings of depression. He admitted to feelings of loneliness due to seclusion from his family who were unable to visit because of COVID-19 isolation parameters. He reported difficulty swallowing, speaking, and increased difficulty eating, due to a combination of dryness in his throat, along with increased feelings of depression. The patient was started on 15mg of Mirtazapine nightly.

**Figure 2:**
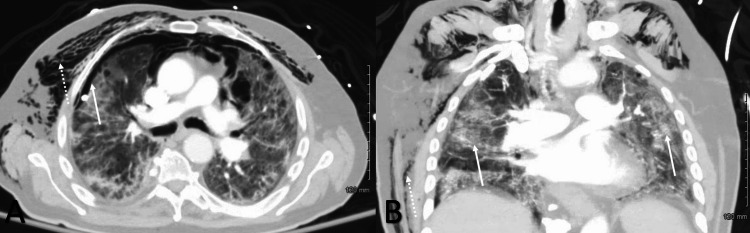
Computed tomography of the thorax with IV contrast A) Transverse computed tomography of the thorax with IV contrast showing soft tissue emphysema on the right and left (noted by the dashed white arrow), worse on the right along with residual small pneumothorax (noted by the solid white arrow). B). Coronal computed tomography atelectasis in bilateral lung spaces indicated with the solid white arrows and subcutaneous emphysema indicated with the dashed white arrow.

On hospital day 16, chest wall crepitus was noted on physical exam and a chest x-ray showed subcutaneous emphysema. A persistent air leak was noted on the chest tube drainage system while the system was on suction. A CT scan with IV contrast of the thorax showed extensive air pockets bilaterally, soft tissue emphysema, worsening right-sided consolidations, and a mild pneumothorax despite having a right-sided pleural catheter in place with the system on suction (Figure 2).

On hospital day 19, the patient had completed all of his COVID-19 treatments and his oxygen saturation had improved to 97% on 3L nasal cannula. His mental status improved and he reported his depression had improved. However, the air leak persisted from the chest tube, and attempts at clamping the chest tube or placing it on a water seal would cause a reoccurrence of his pneumothorax. At this time, the cardiothoracic surgery service was consulted for possible VATS and pleurodesis for the management of a suspected bronchopulmonary fistula. On hospital day 22, after the patient completed his 21 days of COVID-19 isolation, he was taken for a right-sided VATS with 8 grams of talc spread evenly throughout the parietal pleura for chemical pleurodesis and placement of a 32 French chest tube.

**Figure 3:**
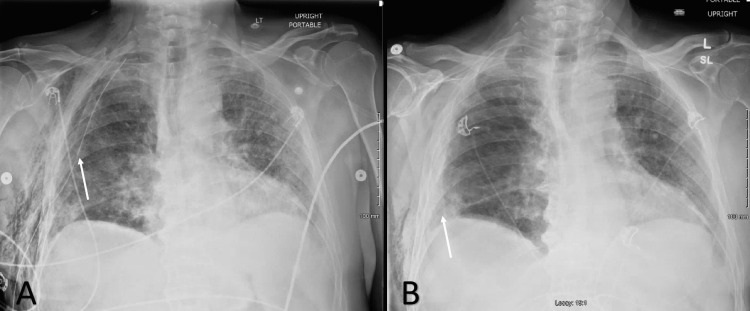
Anterior-Posterior chest x-ray A) Anterior-Posterior chest x-ray on postoperative day one showing no residual pneumothorax and improving atelectasis in the left lung spaces and right lung space with a right-sided chest tube, shown with a white arrow. B). Anterior-Posterior chest x-ray on the day of discharge twenty four hours after the patient’s right-sided chest tube was removed with minor residual lower lung lobe atelectasis indicated with a white arrow.

In the immediate postoperative period, the patient was doing well and had resolution of his pneumothorax while on 20 cm H2O of suction. The chest x-ray taken immediately postoperatively is shown in figure 3A. On postoperative day 11, the chest tube was removed from suction and placed on a water seal; no pneumothorax was noted on the chest x-ray. The patient continued to improve clinically, with improved appetite and caloric intake, increased participation with physical therapy, and resolving feelings of depression. By postoperative day 16, the air leak had resolved, and the chest tube thus was removed. Chest x-ray performed on postoperative day 17 was negative for pneumothorax (Figure 3B). The patient was discharged to a skilled nursing facility on postoperative day 17. On subsequent follow-up, the patient was at home and able to participate in activities of daily living without significant difficulties.

## Discussion

COVID-19 pneumonia can present with a wide spectrum of symptoms. As the clinical course progresses, edema and atelectasis can develop, and worsening of the condition can lead to long-term lung alterations or fibrosis of the parenchyma [5]. Patients exhibit a decreased response to positive end-expiratory pressure, and higher ventilation settings can lead to lung injury, especially in the elderly population [6]. Such insults are a significant reason why barotrauma occurs in 40% of patients with COVID-19 pneumonia who require invasive ventilation.

Bronchopulmonary fistula (BPF) in patients with SARS-CoV-2 pneumonia is a rarely reported complication associated with increased morbidity rates of up to 71% in recent studies [4]. Typically, BPF is seen in the setting of postoperative lung resection; however, other causes include bullous disease, tuberculosis, and radiation therapy [5]. Now, more recently, COVID-19 pneumonia has been identified as another etiology for BPF [7].

Treatment of BPF in a post-operative thoracic surgery patient usually involves repeat thoracotomy with muscle flap closure of the bronchial stump [8]. In non-surgical patients who are often too debilitated for thoracotomy, there are bronchoscopic and pleural procedures that can be utilized. In the current literature, there are various treatment options for a BPF secondary to COVID-19. The overall theme includes minimally invasive techniques including chest tube placements, endobronchial valves, and rarely surgical considerations [7-9]. In this case, a minimally invasive approach utilizing a VATS procedure was successfully used for a debilitated patient suffering from a non-surgical etiology of his BPF. Recent evidence has shown overall the reduced hospitalization and intensive care unit stay in earlier VATS of elderly patients [10]. In this care, simply performing VATS with talc pleurodesis and prolonged chest tube placement on low continuous suction allowed the visceral and parietal pleura to fuse and successfully treat the BPF.

Despite the known risks of invasive surgical treatment of BPF in elderly patients, this case demonstrates how minimally invasive interventions, such as VATS, can repair a BPF. Specifically, VATS was utilized to repair a BPF in an elderly patient suffering from COVID-19 pneumonia in our case. This case report emphasizes that patience and minimally invasive therapy can greatly benefit elderly debilitated patients with sequelae of COVID-19 pneumonia.

## Conclusions

Bronchopulmonary fistula is a serious complication of the SARS-CoV-2 infection, and video-assisted thoracic surgery is one potentially safe and effective surgical option for BPF management in an elderly patient with persistent air leak due to COVID-19 complications. We recommend monitoring the patient postoperatively and recommend for the patient to pass the chest tube clamping trial prior to discharge home. Air leak should also improve to the point where it becomes negligible prior to discharge.
